# Extending Stroke CT Angiography to the Full Chest Allows for the Detection of Additional Pulmonary Opacifications in Acute Stroke Patients

**DOI:** 10.2174/1573405618666220629155250

**Published:** 2025-10-21

**Authors:** Dimah Hasan, Keihan Darvishi, Rebecca May, Hani Ridwan, Alexander Riabikin, Martin Wiesmann, Arno Reich, Omid Nikoubashman

**Affiliations:** 1 Department of Diagnostic and Interventional Neuroradiology, University Hospital RWTH Aachen; Pauwelsstr, 30, 52074 Aachen, Germany; 2 Department of Neurology, University Hospital RWTH Aachen; Pauwelsstr, 30, 52074 Aachen, Germany

**Keywords:** Stroke, COVID-19, Computed tomography angiography, Pulmonary opacity, Scan, Opacifications

## Abstract

**Background and Purpose:**

During epidemics with an increased prevalence of pulmonary infections, extending stroke CTA examinations of acute stroke work up to the whole chest may allow for the identification of pulmonary findings that would have been missed on standard CTA examinations.

**Materials and Methods:**

Our analysis comprised 216 patients with suspicion of stroke who received extended full-chest cerebrovascular CTA examinations from January 27th 2020 - date of the first confirmed Covid-19 case in Germany - until April 30th 2020.

**Results:**

Consolidations and ground-glass opacifications were found in 73 of all 216 patients (34%). Opacifications were found in the upper chest in 51/216 patients (23%). There were lower-chest opacifications in 22 of 165 patients (13%) with unsuspicious upper-chest scans. In these 22 patients, there were consolidations in 10 cases (45%), ground-glass opacifications in 10 cases (45%), and both in 2 cases (10%).

**Conclusion:**

Our study showed that extending the scan volume of an emergency stroke CTA to the whole chest reveals a considerable number of opacifications that would have been missed on a standard CTA. Even though these findings were rarely indicative of COVID-19, a large number of opacifications warranted further investigation.

## 1 INTRODUCTION

Acute ischemic stroke affects mainly the elderly, who are particularly at risk for severe courses of pulmonary infections [1]. The recent coronavirus disease (COVID-19) pandemic made it evident that early identification and treatment of potentially infected patients as well as the early start of protective measures for the surrounding patients and health workers is a key to containment [2].

As acute diagnostic workup and endovascular treatment of stroke patients necessitate interdisciplinary teamwork, not only the patient but also various health workers are at risk, including neurologists, nurses, technicians, neuroradiologists, and anesthesiologists.

While polymerase chain reaction (PCR) tests take up to 24 hours, chest computed tomography (CT) allows immediate diagnosis of pulmonary opacifications. In fact, the sensitivity of chest CT for COVID-19 in early stages is reported to be 56-98% [3, 4].

Diagnostic workup in acute stroke patients includes CT angiography (CTA) of the brain-supplying arteries, commonly including the upper thorax with the aortic arch. Recent studies addressed the importance of additional chest CT for COVID-19 detection in patients with suspected stroke or head trauma [5]. Hence, it could be worthwhile to extend CTA to the full chest to detect pulmonary findings, particularly in times of infectious pandemics. The aim of this study was to evaluate if additional pulmonary opacifications can be found in such an extended CTA compared to a standard CTA, which reaches the aortic arch.

## 2 MATERIALS AND METHODS

### 2.1 Patients

Our hospital is a tertiary stroke center with a catchment area of approximately 1,000,000 inhabitants in our own district and our two referring neighboring districts. The first documented case of COVID-19 in our catchment area occurred in our neighboring district, which became one of the most heavily affected in the nation, on February 25th. The cumulated number of COVID-19 cases per 100,000 inhabitants in our district and our two referring neighboring districts increased slowly from 6.9 on March 1st to 79.0 on March 15th and then increased more steeply with 391.5 cases on April 30th, and finally 415.4 cases on May 31st.

For our retrospective analysis, we searched our medical database for patients with suspicion of stroke who received CTA from January 27th 2020, the date of the first documented case of COVID-19 in Germany, until April 30th 2020. We identified 296 consecutive patients and excluded 80 patients, in whom CTA did not include the full chest. This left 216 patients to be included in our study. To adjust for the increasing prevalence of pulmonary infections during different phases of the epidemic, we compared CT scans before the steep increase of infections (January 27th to March 15th, “first phase”) and after the beginning of the steep increase (March 16^th^ to April 30^th^, “second phase”).

Our local ethics committee approved this analysis.

### 2.2 Imaging

All patients were examined on a 40-slice CT scanner (Somatom Volume Zoom; Siemens Medical Solutions, Erlangen, Germany). Our standard acute stroke protocol includes a spiral CTA from the distal cranium to the diaphragm with a reconstructed section width of 0.6 mm. A 100-mL dose of nonionic contrast agent with an iodine concentration of 300 mg/mL (Solutrast 300, Bracco Imaging GmbH, Konstanz, Germany) and a 20-mL dose of isotonic saline were administered bolus–triggered in a cubital vein with a flow rate of 3 mL/s. The patients’ arms were always positioned downwards, parallel to the chest. We used a breath command in all CTA exams after 10th of March. From the CTA datasets, we constructed an axial lung-window chest CT with a 3 mm reconstruction width.

**Fig. (1):**
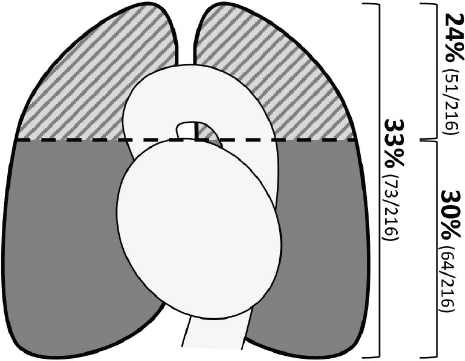
Illustration of scan areas of a standard stroke CT angiography (upper area) and our extended CT angiography, including the whole chest. Numbers indicate the number of pulmonary opacifications per area.

For this analysis, we focused on the presence of opacifications 1) in the upper chest containing the aortic arch (“standard CTA”) and 2) the lower chest (“extended CTA”) (Fig. **1**). Pulmonary opacifications were categorized as 1) consolidations or 2) ground glass opacifications. Opacifications were further categorized according to the CO-RADS scheme, with category 4 and 5 opacifications being defined as highly suspicious for COVID-19 [6]. Other pulmonary changes such as dystelectasis and pleural effusions were categorized as “other” and not classified as opacifications. All imaging data were reviewed by two independent radiologists, and a reference standard for statistical analysis was established in a consensus reading.

Radiation dose was compared using dose length products (DLP) of an internal quality control sample with samples of 20 patients each from 1) a historical cohort of our patients with CTA reaching the aortic arch, 2) the recent cohort of patients with full chest CTA, 3) and a cohort of dedicated non-contrast Covid-19 chest CT exams.

### 2.3 Statistical Analysis

Symmetrically distributed variables were described with means and standard deviations, while skewed variables were described by the median (IQR). We used Student’s t-tests and χ2 tests depending on sample type. *P* values with an α-level < 0.05 were defined as significant. All statistical analyses were performed with SPSS 25 software.

## 3 RESULTS

The median age of our patients was 73 years (IQR, 63-82) and 93/216 patients (43%) were female. We examined 104 patients at the beginning of the epidemic and 112 patients in the following phase.

Pulmonary findings of any sort, including consolidations, ground-glass opacifications, dystelectasis, and pleural effusion, were present in 180/216 patients (83%). There were pulmonary findings in the upper chest in 109/216 patients (50%). In 71/107 patients (66%) with normal upper-chest exams, there were pulmonary findings of any sort in the lower chest. Consolidations and ground-glass opacifications, which are suspicious for infection, were found in only 12/107 patients (11%) with normal upper-chest exams.

### 3.1 Consolidative and Ground-glass Opacities

Overall, pulmonary opacifications defined as consolidations and ground-glass opacifications were found in 73 of all 216 patients (33%) Fig. (**1**) Findings were highly suspicious for COVID-19 in 9 of these 73 patients (13%). There was no significant difference in the overall number of opacifications during the beginning of the epidemic and the second phase, with opacifications being found in 29% (30/104) in the first phase and 38% (43/112) in the second phase (p=.138). However, opacifications that were highly suspicious for COVID-19 (CO-RADS 4&5) were significantly more often in the second phase, with 1% (1/104) in the first phase and 7% (8/112) in the second phase (p=.023).

Opacifications in the upper chest were found in 51 of all 216 patients (24%) Fig. (**1**). Of the other 165 patients with unsuspicious upper thorax scans, 22 patients (13%) had opacifications in the lower chest Fig. (**2**). In these 22 patients, there were consolidations in 10/22 cases (45%), ground-glass opacifications in 10/22 cases (45%), and both in 2/22 cases (10%). Nineteen of these 22 patients (86%) were asymptomatic at time of imaging.

There was no significant difference between the beginning of the epidemic and the second phase: There were lower-chest opacifications in patients with unsuspicious upper-chest exams in 12% (10/84) in the first phase and 15% (12/81) in the second phase (p=.583). None of these 22 patients had findings that were highly suspicious for COVID-19.

### 3.2 PCR

Overall, 51 of all 216 patients (24%) were tested for COVID-19, of whom 5/51 patients (10%) were PCR-positive. Four of the latter had highly suspicious opacifications on chest CT, whereas the remaining patient had a normal CT scan. All 4 patients with positive PCR also had opacifications in the upper parts of the chest.

### 3.3 Radiation

Mean DLP was 228±43 mGy*cm for aortic arch CTA exams, 289±32 mGy*cm for full chest CTA exams, and 144±51 mGy*cm for dedicated COVID-19 chest CT exams. DLP of full chest CTA exams was significantly higher than that of aortic arch CTA exams (p<.0001).

## 4 DISCUSSION

Our study showed that extending the scan volume of an emergency stroke CTA to the whole chest reveals a considerable number of opacifications that would have been missed on a standard CTA. Even though these findings were rarely indicative of COVID-19, a large number of opacifications warranted further investigation. In fact, consolidations and ground-glass opacifications would have been missed in every 7th patient (13%) if CTA had reached the aortic arch only. As the majority of these patients were asymptomatic at the time of imaging, CTA allowed for anticipation of clinical/preventive measures before patients were symptomatic.

Recent studies showed that abdominal or head/neck-CT may reveal the first signs of COVID-19 infection in the lower or upper thorax, respectively, in spite of missing respiratory symptoms [7, 8].

With reference to stroke CTA, earlier studies during the COVID-19 pandemic showed that the presence or absence of ground glass opacity in lung apices is a reliable and accurate biomarker for the diagnosis of COVID-19 [8, 9]. However, the stroke CTA in these studies was not extended to the region under the aortic arch, which may result in missing the diagnosis in many cases considering the previously described distribution patterns of infiltrates in COVID-19 pneumonia [4, 10]. Interestingly, the severity of lung disease in COVID-19 as scored on lung CT was also found to be predictive of acute abnormalities on neuroimaging in patients with COVID-19 with neurologic manifestations. Thus, lung CT findings can be used as a predictive tool in patient management to improve clinical outcomes [11].

The increasing incidence of COVID-19-suspicious findings with increasing prevalence in the general population implies that an extended CTA is particularly of use when the prevalence of pulmonary infections is high. As pulmonary opacifications in the lower chest would have been missed in 12% of cases at the beginning of the epidemic, it is worthwhile to investigate whether the benefits of an extended CTA might justify additional radiation exposure even outside of epidemic times.

Concerning radiation dose, extending the CTA to the full chest resulted in a significant increase of DLP by approximately 27%. However, the additional average DLP of 61 mGy*cm was less than half as high than that of a dedicated COVID-19 chest CT. In summary, our results show that it is worthwhile to extend acute stroke CTA exams to the whole chest. This was even the case, although conditions were not ideal, with the scan protocol not being optimized for a chest CT, arms being positioned next to the body, and breath commands being introduced inconsistently.

## 5 LIMITATIONS

Our study has some limitations which need to be addressed. First of all, not all patients were tested for COVID-19 using PCR, but only patients with symptoms, history of contact with other COVID-19 patients or suspected CT. However, this finding allows concluding that extending stroke CTA to the whole thorax elevates the sensitivity of detecting COVID 19 infection with low additional radiation exposure, which could facilitate more infection control. Secondly, the study is retrospective and does not allow to determine causation of the findings. However, the association of stroke and highly prevalent sub-aortal opacification in the extended CTA in the era of the COVID-19 pandemic is a very important finding which has to be further studied.

## CONCLUSION

Our study showed that extending the scan volume of an emergency stroke CTA to the whole chest reveals a considerable number of opacifications that would have been missed on a standard CTA. Even though these findings were rarely indicative of COVID-19, a large number of opacifications warranted further investigation. Our results imply that the benefits of an extended CTA in stroke patients may justify the additional radiation exposure, particularly as additional DLP of an extended CTA is almost only half as high than DLP of a dedicated low-dose chest CT.

## Figures and Tables

**Fig. (2):**
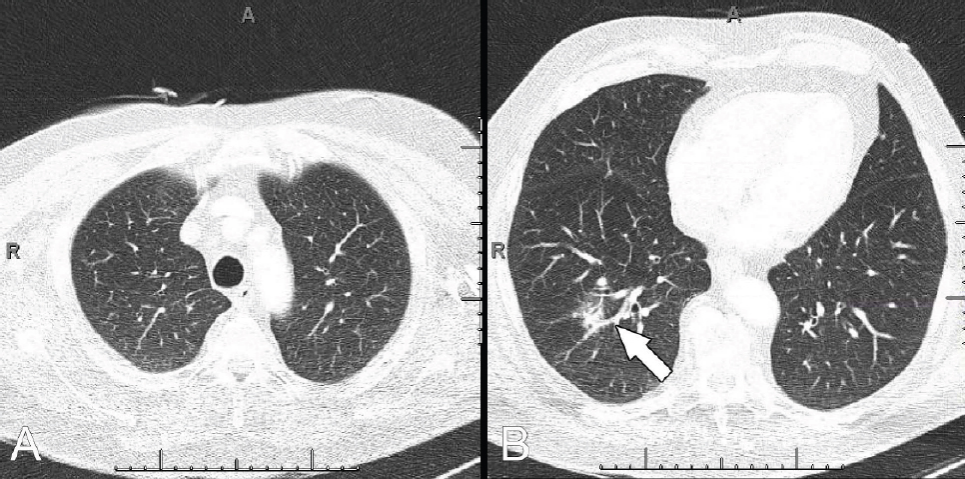
Lung-window chest CT of a stroke CT angiography in a patient with an unsuspicious upper-chest exam (**A**) and opacifications in the lower parts (**B**: arrow) (CO-RADS 3).

## Data Availability

Not applicable.

## LIST OF ABBREVIATIONS

COVID-19: Coronavirus Disease
CT: Computed Tomography
CTA: CT Angiography
PCR: Polymerase Chain Reaction

## STANDARDS OF REPORTING

STROBE guidelines were followed.
